# Genetic and autoimmune predispositions to fulminant viral hepatitis in children

**DOI:** 10.3389/fimmu.2026.1858895

**Published:** 2026-08-07

**Authors:** Mohamed Bousfiha, Dalal Ben Sabbahia, Emmanuelle Jouanguy, Jean-Laurent Casanova, Jalila El Bakkouri, Ahmed Aziz Bousfiha, Naima Amenzoui

**Affiliations:** 1 Laboratory of Clinical Immunology, Infection and Autoimmunity, Faculty of Medicine and Pharmacy of Casablanca, Hassan II University of Casablanca, Casablanca, Morocco; 2 Department of Clinical Immunology and Pediatrics Infectious Diseases, Abderrahim El Harouchi Mother-Child Hospital, Ibn Rochd University Hospital Center, Casablanca, Morocco; 3 Pediatric Gastroenterology Unit, Pediatrics 3, Ibn Rochd University Hospital Center, Casablanca, Morocco; 4 Laboratory of Human Genetics of Infectious Diseases, Necker Branch, Institut National de la Sante et de la Recherche Medicale (INSERM) U1163, Paris, France; 5 Paris Cité University, Imagine Institute, Paris, France; 6 Laboratory of Human Genetics of Infectious Diseases, Rockefeller Branch, Rockefeller University, New York, NY, United States

**Keywords:** fulminant viral hepatitis, IL-10RB deficiency, IL-18BP deficiency, inborn errors of immunity, type I interferon autoantibodies

## Abstract

Fulminant viral hepatitis (FVH) in children is a rare but often fatal form of acute liver failure occurring in the absence of preexisting liver disease. Its exceptional incidence during otherwise common viral infections, including hepatitis A virus (HAV), hepatitis B virus (HBV), and herpes simplex virus (HSV), supports a decisive role for host susceptibility. Recent advances in human immunogenetics delineate two major, mechanistically distinct pathways to pediatric FVH. The first reflects failure of immune regulation, culminating in excessive IFN-γ–driven inflammation and immune-mediated hepatocellular necrosis. Autosomal recessive IL-18BP and IL-10RB deficiencies exemplify this mechanism, in which disruption of key regulatory checkpoints permits uncontrolled activation of cytotoxic lymphocytes and macrophage-dependent immunopathology, particularly in the context of HAV infection. The second pathway involves impaired intrinsic antiviral defense, most prominently through neutralizing autoantibodies against type I interferons, which phenocopy genetic defects of IFN-I signaling and are strongly associated with HSV-triggered FVH; in this setting, inadequate early antiviral control enables unchecked hepatic replication with extensive cytopathic damage. Finally, syndromic hyperinflammatory disorders, including familial hemophagocytic lymphohistiocytosis and X-linked lymphoproliferative disease, broaden the spectrum of immune predisposition in which fulminant hepatitis may arise. Together, these discoveries redefine pediatric FVH as an immunopathological syndrome and provide a framework for targeted genetic and serologic diagnosis and for mechanism-based interventions aimed at improving survival.

## 1 Introduction

Fulminant viral hepatitis (FVH) is a rare form of acute liver failure triggered by viral infection, characterized by rapid hepatocellular necrosis, coagulopathy, and encephalopathy in individuals without prior liver disease (1, 2). It most commonly follows primary infection with hepatitis A or B virus (HAV or HBV), although other viruses, such as herpes simplex virus (HSV), can also act as triggers, though less frequently (1). The incidence of FVH is exceedingly low, on the order of one to five cases per million infections, and only a tiny fraction of symptomatic hepatitis cases progress to fulminant liver failure, accounting for <0.5% for HAV and ~0.1% for HBV in unvaccinated children (3, 4). Despite its rarity, FVH carries a poor prognosis: in the absence of liver transplantation, survival is typically below 20%, whereas timely transplantation can increase survival to approximately 80% (4, 5).

The pathogenesis of FVH has long remained poorly understood. Its sporadic occurrence, even with typical viral strains, indicates that viral factors alone cannot explain this extreme clinical phenotype, pointing instead to inter-individual differences in host immunity as a key determinant of disease outcome (1). Two principal mechanisms of host predisposition have emerged over the past decade (6–9). First, inborn errors of immunity (IEI), illustrated by autosomal recessive (AR) IL-18 binding protein (IL-18BP) deficiency and IL-10 receptor β (IL-10RB) deficiency, disrupt immune regulatory pathways, leading to uncontrolled inflammation and hepatocyte necrosis (6–8). Second, acquired autoimmune mechanisms, most clearly neutralizing autoantibodies against type I interferons (IFN-α and IFN-ω), impair antiviral defense, permitting unrestrained viral replication and liver injury (9).

These pathways encompass both isolated FVH, in which fulminant hepatitis is the leading manifestation, as demonstrated in autosomal recessive IL-18BP deficiency (6, 7), and syndromic FVH occurring within broader immune disorders, such as IL-10RB deficiency with very-early-onset inflammatory bowel disease (8), and the HLH and XLP spectrum, in which fulminant hepatitis may occur in the setting of systemic hyperinflammation and lymphoproliferation (10–12). In addition, inborn errors affecting type I interferon dependent antiviral immunity, from pathogen sensing to IFNAR-STAT signaling, have been implicated in severe organ-restricted viral disease (13–16), with only one reported case of fulminant hepatitis of unidentified etiology in a STAT2-deficient child (13). Acquired neutralizing autoantibodies against type I IFNs represent a broader and better-documented mechanism of IFN-I insufficiency leading to fulminant hepatitis (9).

This review examines the genetic and autoimmune mechanisms that predispose children to fulminant viral hepatitis. We outline the immunopathology of these pathways, emphasizing how disruptions in immune regulation or antiviral defense converge to cause severe liver injury. Furthermore, we discuss clinical implications, including approaches for identifying at-risk patients and potential targeted interventions, such as cytokine replacement therapy or exogenous interferon administration, which may guide personalized management strategies.

## 2 Inborn errors of immunity predisposing to FVH

### 2.1 IL-18BP deficiency

The first monogenic etiology of FVH was discovered in 2019 by the identification of a biallelic loss-of-function mutation in *IL18BP* in an 11-year-old girl who died from fulminant HAV infection (6). *IL18BP* encodes IL-18 binding protein, a natural antagonist of the proinflammatory cytokine IL-18. In a healthy immune response to HAV, IL-18 is secreted by hepatic macrophages to activate lymphocytes, inducing interferon-gamma (IFN-γ) production and cytotoxicity to kill infected hepatocytes; concurrently, IL-18BP secretion, by hepatocytes and macrophages, is induced by IFN-γ to neutralize IL-18 activity. In IL-18BP–deficient patients, this braking mechanism is lost. As a result, IL-18 activity is not regulated, leading to excessive IFN-γ production and uncontrolled immune-mediated hepatotoxicity (6). This IFN-γ-mediated inflammation is directly toxic to the liver; indeed, it provides proof of principle that a single-gene defect unleashing IL-18 can cause fulminant hepatitis. Subsequent reports have confirmed IL-18BP deficiency as a *bona fide* genetic etiology of fulminant HAV hepatitis: for example, two Egyptian siblings with a novel IL18BP loss-of-function mutation both succumbed to HAV-FVH (7). Intriguingly, those IL-18BP–deficient children remained resistant to other common infections, and a third sibling survived non-HAV hepatitis (CMV/EBV), suggesting IL-18BP is specifically essential for controlling HAV-induced liver inflammation (6, 7).

### 2.2 IL-10RB deficiency

Another inborn immunodeficiency discovered in children with fulminant hepatitis is autosomal recessive IL-10 receptor beta chain (IL-10RB) deficiency (8). IL-10RB is a subunit of the IL-10 receptor, critical for signaling of the anti-inflammatory cytokine IL-10 as well as IL-22, IL-26, and type III interferons (IFN-λ). In 2023, two siblings who died of fulminant HAV infection at ages 6 and 1, were found to have inherited IL-10RB deficiency, establishing it as a genetic cause of FVH (8). Pathophysiologically, IL-10 normally serves as a brake on macrophage activation and IFN-γ production; therefore, loss of IL-10 signaling leads to uncontrolled IFN-γ-mediated inflammation, very similar to the IL-18BP scenario. Indeed, both IL-18BP and IL-10RB deficiencies converge on the same outcome: excessive IFN-γ activity in the liver, driving a hyperinflammatory, necrotoxic response to the virus (8). Of note, both siblings also suffered from severe infantile inflammatory bowel disease (IBD), consistent with the known phenotype of complete IL-10/IL-10R deficiencies (8). However, the clinical phenotype in these siblings reveals FVH as an unrecognized risk in IL-10 pathway defects. This highlights that patients with IL-10 signaling deficiencies should be recognized as immunodeficient not only in the gastrointestinal tract but also in the hepatic context, warranting proactive measures, such as HAV vaccination.

### 2.3 Other genetic susceptibility factors

#### 2.3.1 Hemophagocytic lymphohistiocytosis and related genetic syndromes

Severe hepatic injury resembling FVH can also occur in the setting of genetic disorders that drive an overly aggressive immune response to viral infection. The prototype is familial hemophagocytic lymphohistiocytosis (FHL), a group of autosomal recessive defects (mutations in *PRF1*, *UNC13D*, *STXBP2*, etc.) where cytotoxic T and NK cells cannot properly kill their targets, leading to persistent immune cell activation and overwhelming cytokine release (IFN-γ, TNF, etc.), driving severe hyperinflammation and multiorgan damage. A classic trigger is Epstein–Barr virus (EBV): in patients with perforin deficiency (*PRF1* mutations) or other FHL genes, EBV infection frequently leads to fulminant HLH with hepatic involvement, coagulopathy, and multi-organ failure, driven by systemic hemophagocytic infiltration rather than primary viral hepatic injury (17, 18). Similarly, X-linked lymphoproliferative disease (XLP) exemplifies how a single-gene immune defect can cause fatal hepatic disease in the context of uncontrolled viral infection. XLP type 1, due to mutations in *SH2D1A* (which encodes the SAP adaptor protein), is characterized by an inability to control EBV; affected boys develop uncontrolled T- and B-cell proliferation upon EBV infection, resulting in fulminant infectious mononucleosis and HLH with immune-mediated liver necrosis (10, 19). In fact, EBV-triggered HLH is the hallmark presentation of XLP1, and the original XLP patient registry in the 1980s found a ~75% mortality rate primarily due to hepatic involvement and multi-organ failure following EBV infection (11). XLP type 2 (due to *XIAP* mutations) similarly confers extreme susceptibility to EBV-driven HLH and liver failure (12). Clinically, children with HLH or XLP may present with acute liver failure that closely mimics FVH, though the underlying mechanism is immune-mediated hepatic infiltration rather than direct viral cytopathic injury; systemic features such as fever, cytopenias, and splenomegaly typically point toward the correct diagnosis of HLH rather than primary FVH (10). Early genetic diagnosis in such cases is crucial, as aggressive immunosuppressive therapy or transplant can be life-saving if instituted before the cytokine storm irreversibly damages organs.

#### 2.3.2 Inborn errors of type I interferon immunity

In contrast to the above hyperinflammatory disorders, another group of IEIs predisposes to severe viral disease via the opposite mechanism: an inadequate intrinsic antiviral response. Type I interferons (IFN-α/β) are pivotal for early control of most viruses, including herpesviruses and hepatotropic viruses. Rare germline mutations that abolish IFN-I signaling have been described, such as autosomal recessive STAT2 deficiency or IFN-α/β receptor (*IFNAR1/2*) deficiencies; these conditions render patients susceptible to severe adverse reactions to live attenuated viral vaccines and to unusually severe wild-type viral infections (13–15), including HSV encephalitis (16). Direct evidence linking these genetic defects to fulminant viral hepatitis specifically remains limited: in a 2023 multicenter study of 23 STAT2-deficient patients, one 5-year-old child died from fulminant hepatitis of unidentified etiology, suggesting that complete loss of IFN-I signaling may permit uncontrolled intrahepatic viral replication and massive cytopathic damage (13). However, no viral trigger was confirmed in this case (13), and frank FVH has not been documented among IFNAR1/2-deficient patients (14–16). Importantly, unlike IL-18BP or IL-10RB defects which drive excess IFN-γ, defects in type I IFN pathways result in interferon insufficiency, and the immunopathology in such cases likely reflects a high viral burden triggering widespread hepatocyte death rather than immune-mediated injury. Consistent with this, some STAT2-deficient patients developed severe adenovirus or enterovirus hepatitis with pneumonia in the absence of classical HLH (13).

#### 2.3.3 Genetic associations

Beyond high-penetrance monogenic disorders, researchers have explored whether more common genetic variants might influence FVH risk. Genome association studies have hinted at a few such polymorphisms (20, 21). For example, a variant in *TIM1* (HAVCR1), encoding a receptor for HAV, was associated with increased susceptibility to severe HAV infection. One study found a particular *TIM1* polymorphism raised the odds of fulminant hepatitis A by ~1.3-fold (20). Similarly, a *CXCL16* gene variant was linked to acute liver failure in HBV infection (about 1.6-fold risk) (21). While statistically significant, these common variants confer only modest risk, far smaller than the effect of rare monogenic IEIs, and it is likely that FVH in some patients may result from an unfortunate combination of such polygenic risk factors and environmental triggers. By contrast, a biallelic *IL18BP* or *IL10RB* mutation virtually guarantees severe HAV-FVH if infected. There is also evidence that other viruses’ fulminant courses may have genetic contributors: a recent study of 24 patients with fulminant hepatitis E found that 10 patients harbored rare variants in genes involved in intrinsic antiviral immunity (particularly the type I interferon pathway), not seen in controls (22). Likewise, familial clustering of fulminant HBV or other severe viral hepatitis cases is occasionally noted, hinting at undiscovered IEIs. In summary, while polygenic risk factors (like *TIM1* or *CXCL16* variants) may slightly tilt the odds toward severe disease, high-impact single-gene defects in immune regulation, as well as their autoimmune phenocopies, have emerged as the clearest genetic causes of pediatric FVH identified to date.

## 3 Autoimmune predispositions to FVH

In addition to germline mutations, an emerging body of evidence shows that acquired autoimmune factors, specifically, autoantibodies against key antiviral cytokines, can predispose patients to severe viral infections. This mechanism essentially creates an *acquired immunodeficiency*, or a “phenocopy” of an inborn error. Such autoantibodies have come to prominence in other life-threatening viral illnesses, such as anti–IFN-α antibodies in severe COVID-19 pneumonia or arboviruses (23–25). Recently, Gervais et al. reported that a significant subset of children and adults with HSV-triggered FVH harbored pre-existing neutralizing autoantibodies to type I interferons (9). In their international cohort, 6 of 16 patients (~37.5%) with fulminant HSV hepatitis had anti–type I IFN auto-Abs, whereas none of 133 patients with HAV- or HBV-induced FVH had such antibodies (9). These auto-Abs were detected in the bloodstream at hospital admission, indicating they were present before the acute infection. Functionally, the autoantibodies completely blunt type I IFN activity, disabling a critical antiviral mechanism and permitting rampant HSV replication in the liver (9). Notably, the presence of these auto-Abs was associated with astronomically high odds ratios for developing HSV-FVH, ranging from ~35 for low-level neutralization to nearly 1,900 in patients whose auto-Abs neutralized IFN-α at high concentrations (9). Thus, autoantibodies against type I IFNs appear to be a major determinant of fulminant HSV hepatitis susceptibility.

## 4 Discussion

Recent human genetics studies have revealed that pediatric fulminant viral hepatitis (FVH) often reflects two fundamentally different immune failure modes rather than a single disease process. In one path, genetic defects in immune regulation unleash an overactive immune attack on the liver. For example, autosomal recessive *IL18BP* loss-of-function prevents neutralization of IL-18, resulting in a massive IFN-γ production and immune mediated-hepatotoxicity (6, 7). Belkaya et al. demonstrated that an IL-18BP–deficient child developed HAV-induced FVH (6), a finding later corroborated by the description of two additional children with HAV-FVH caused by autosomal recessive IL18BP deficiency (7), establishing IL-18BP deficiency as a genetic cause of HAV-FVH. Similarly, loss of IL-10 signaling, such as IL10RB mutations, remove a key anti-inflammatory checkpoint, allowing unchecked macrophage activation and IFN-γ–driven liver injury (8). Korol et al. described siblings with IL-10RB deficiency who died from HAV-FVH, highlighting the non-redundant role of IL-10 in restraining hepatic inflammation (8). These genetic disorders resemble HLH or macrophage activation syndrome, with extreme hyperferritinemia, cytokine excess, and dense hepatic infiltration by activated T cells, NK cells, and macrophages. Indeed, classic familial HLH or X-linked lymphoproliferative disorders (e.g. SH2D1A/XLP1) can present with severe hepatic injury and features mimicking fulminant hepatitis during viral triggers (especially EBV), though the underlying mechanism is immune-mediated hepatocellular destruction driven by uncontrolled cytotoxic T-cell and macrophage activation rather than direct viral cytopathic injury, underscoring that excessive IFN-γ–mediated immunity can devastate the liver (10, 11).

By contrast, the other FVH pathway is an innate immunodeficiency permitting rampant viral replication (9). The clearest example is the presence of neutralizing autoantibodies against IFN-α/ω in patients with HSV‐triggered FVH (9). In a cohort of 149 FVH cases, Gervais et al. found that ~37.5% of HSV‐FVH patients carried high-titer anti–IFN-α/ω antibodies, whereas none of the HAV- or HBV-induced cases did (9). These autoantibodies functionally phenocopy an inherited IFN-I signaling defect: without type I IFN activity, herpes simplex virus can replicate unchecked in hepatocytes. The liver injury is thus initially driven by viral cytopathic effects, often followed by a secondary inflammatory reaction. In effect, these patients often exhibit extremely high HSV viral loads and poor response to antivirals (9), allowing direct HSV-mediated lysis of hepatocytes, in contrast to the immune-mediated injury of the first route (6–8). Of note, a patient with complete STAT2 deficiency died from fulminant hepatitis of unidentified etiology, consistent with this mechanism, although frank FVH has not been reported in other IFN-I pathway defects to date (13).

These two pathways appear to be largely virus-specific and mutually exclusive. Genetic IFN-γ–hyperactivation disorders predispose primarily to non-cytopathic hepatotropic viruses (HAV, HBV, EBV), whereas type I IFN deficiencies (genetic or autoimmune) lead almost exclusively to HSV hepatitis. Indeed, patients with anti–IFN-I autoantibodies have no reported cases of HAV-FVH (9). Intriguingly, no patient has been described with both HSV encephalitis and HSV-FVH, despite both being linked to IFN-I immunity (9). This suggests the existence of organ-specific immune redundancies or compartmentalized antiviral defenses, a concept now emerging in immunogenetics (9). Similarly, patients with inborn IFN-I pathway deficiencies are known to be extraordinarily susceptible to herpesviruses, like HSV or varicella (e.g. STAT1, TLR3, or IRF3 mutations famously cause HSV encephalitis), but they do not generally get worse disease from HAV or HBV (14–16). Consistently, none of the STAT2-deficient patients had unusual severity with HAV or HBV; rather, the concern was severe herpesvirus or other systemic viral infections (13). Fulminant hepatitis thus lies on a spectrum from “hyper-immune” to “hypo-immune” extremes. Each mechanism converges on the same catastrophic outcome – massive hepatocyte death – but the underlying immunopathology and viral triggers differ (**Table 1**).

**Table 1:** Key differences between monogenic (genetic) vs. autoimmune predispositions to fulminant viral hepatitis.

Feature	Genetic predisposition (monogenic IEI, hyperinflammatory)	Autoimmune predisposition (anti–cytokine autoantibodies)
Underlying defect	Inherited monogenic errors in immune-regulatory genes (e.g. IL18BP, IL10RB, FHLH genes)	Acquired neutralizing auto-Abs against cytokines (e.g. IFN-α/ω)
Immune axis affected	Type II IFN pathway (IL-18 IL-10/IFN-γ) dysregulation	Type I IFN pathway (IFN-α/β/ω) blockade
Mechanism of injury	Excessive inflammatory response and cytotoxicity (cytokine storm)	Impaired early antiviral control → high viral load and direct cytopathy
Typical viral triggers	Hepatotropic viruses (HAV, HBV; also EBV in HLH/XLP syndromes)	Herpesviruses (HSV-1/2)
Example conditions	IL-18BP or IL-10RB deficiency; familial HLH (PRF1, etc.)	Auto-Abs to IFN-α/ω (seen in APS-1 and some FVH patients)

This conceptual framework has practical implications. Clinicians should consider these mechanisms when evaluating a child with cryptogenic acute liver failure. For instance, HSV-FVH should prompt testing for anti–IFN-I autoantibodies, whereas unexplained severe HAV hepatitis may warrant genetic screening. Importantly, understanding the mechanism suggests targeted therapies. In hyperinflammatory cases (e.g. IL-18BP/IL-10RB defects), immunomodulation (corticosteroids, anti–IFN-γ antibodies) or cytokine replacement (recombinant IL-18BP) could mitigate the immune storm. In contrast, cases of IFN-blockade–mediated FVH may benefit from antiviral strategies and immune therapies (e.g. plasmapheresis to remove autoantibodies, or administration of alternate interferons like IFN-β/λ) to restore antiviral defense. Thus, FVH is no longer an idiopathic disease but a syndrome that can, in many instances, be explained mechanistically and potentially treated with precision approaches.

## 5 Conclusion

Fulminant viral hepatitis is increasingly understood not as a consequence of an unusually virulent pathogen, but as the striking manifestation of a host immune defect. Recent discoveries have shown that FVH often reflects either excessive immune activation or a critically insufficient one. On one hand, monogenic errors, such as IL-18BP, IL-10/IL-10R deficiencies (or familial HLH mutations), unleash an unrestrained IFN-γ–mediated inflammatory storm that destroys the liver during a routine hepatitis A/B/EBV infection. On the other hand, acquired anti–type I IFN autoantibodies (or related genetic defects like STAT2 or IFNAR mutations) abrogate the first line of defense, allowing viruses such as HSV to replicate unchecked and cause devastating cytopathic injury. Thus, the same clinical outcome, fulminant hepatitis, can result from either excessive or insufficient immune responses.

Recognizing these patterns allows clinicians to identify at-risk children (through family history or autoimmune features), to confirm diagnoses with genetic or serologic tests, and to intervene in novel ways. For instance, a child with IL-10RB deficiency should be vaccinated against HAV before exposure, and a patient with anti-IFN autoantibodies might receive alternative interferon therapy during HSV infection. Continued research is likely to uncover additional immune causes of FVH and to refine treatment strategies. The goal is to transform FVH from a cryptic, high-mortality syndrome into a situation where preventive and targeted therapies can improve survival. In summary, the extreme outcomes of pediatric FVH are often the predictable result of underlying immune imbalances, and by understanding these hidden susceptibilities, we can move toward preventing and treating what was once an unexplained tragedy.
